# Supplementary material for: Microstimulation of visual area V4 improves visual stimulus detection
Source: Cell Rep. 2022 Sep 20;40(12):111392. doi: 10.1016/j.celrep.2022.111392 (PMC9513802; doi:10.1016/j.celrep.2022.111392)
Supplement: Document S1. Tables S1 and S2 [file mmc1.pdf]

Cell Reports, Volume *40*

Supplemental information

Microstimulation of visual area V4 improves visual stimulus detection

Ricardo Kienitz, Kleopatra Kouroupaki, and Michael C. Schmid

Supplementary Information

Catch trial performance				
	No stim		Stim	
	in-RF	out-RF	in-RF	out-RF
Monkey K	97.1%	92.3%	98.1%	86.3%
Monkey H	97.9%	90.3%	97.5%	91.3%
Change of fixation breaks with microstimulation				
	in-RF	p	out-RF	p
Monkey K	21.4%	0.38	-8.6%	0.67
Monkey H	28.6%	0.67	5.6 %	0.89

Table S1. Additional control analyses, related to Figure 1.
Note that fixation breaks showed no consisted or significant change with microstimulation (Chi-square test).

Thresholds – in-RF									
		SOA 0 ms		SOA 200 ms		SOA 400 ms		SOA 600 ms	
	No stim	Stim	p	Stim	p	Stim	p	Stim	p
Monkey K	5.44 ± 0.19	6.34 ± 0.74	0.064	3.22 ± 0.16	1.1×10^{-16}	3.07 ± 0.34	1.9×10^{-9}	2.72 ± 0.17	1.1×10^{-16}
Monkey H	5.53 ± 0.12	4.95 ± 0.42	0.113	4.82 ± 0.62	0.111	3.64 ± 0.39	1.5×10^{-7}	3.74 ± 0.15	1.1×10^{-16}
Thresholds – out-RF									
		SOA 0 ms		SOA 200 ms		SOA 400 ms		SOA 600 ms	
	No stim	Stim	p	Stim	p	Stim	p	Stim	p
Monkey K	4.85 ± 0.20	4.8345 ± 0.40	0.975	2.74 ± 0.21	7.4×10^{-11}	2.65 ± 0.29	8.6×10^{-9}	2.41 ± 0.21	8.8×10^{-14}
Monkey H	5.43 ± 0.19	4.84 ± 0.18	0.061	3.04 ± 0.28	2.2×10^{-11}	3.69 ± 0.35	4.2×10^{-5}	3.76 ± 0.52	3.6×10^{-5}
Slopes – in-RF									
		SOA 0 ms		SOA 200 ms		SOA 400 ms		SOA 600 ms	
	No stim	Stim	p	Stim	p	Stim	p	Stim	p
Monkey K	75.59 ± 10.51	54.42 ± 18.57	0.059	188.12 ± 44.59	5.8×10^{-5}	67.17 ± 24.66	0.544	179.57 ± 50.18	0.0001
Monkey H	143.54 ± 15.44	83.38 ± 36.66	0.087	52.87 ± 25.02	1.5×10^{-8}	84.09 ± 40.32	0.004	311.26 ± 75.02	0.006
Slopes – out-RF									
		SOA 0 ms		SOA 200 ms		SOA 400 ms		SOA 600 ms	
	No stim	Stim	p	Stim	p	Stim	p	Stim	p
Monkey K	55.06 ± 8.51	65.49 ± 19.97	0.353	110.35 ± 24.48	0.002	80.21 ± 29.68	0.115	123.36 ± 26.89	0.002
Monkey H	85.14 ± 12.63	115.07 ± 25.54	0.109	80.36 ± 43.89	0.771	62.18 ± 18.21	0.079	73.94 ± 35.69	0.473

Table S2. Changes of threshold and slope as a function of microstimulation-target SOA, related to Figure 3.
