# Supplementary material for: Microstimulation of visual area V4 improves visual stimulus detection
Source: Cell Rep. 2022 Sep 20;40(12):111392. doi: 10.1016/j.celrep.2022.111392 (PMC9513802; doi:10.1016/j.celrep.2022.111392)
Supplement: Document S2. Article plus supplemental information [file mmc2.pdf]

Cell Reports

Report

Microstimulation of visual area V4 improves visual stimulus detection

Graphical abstract

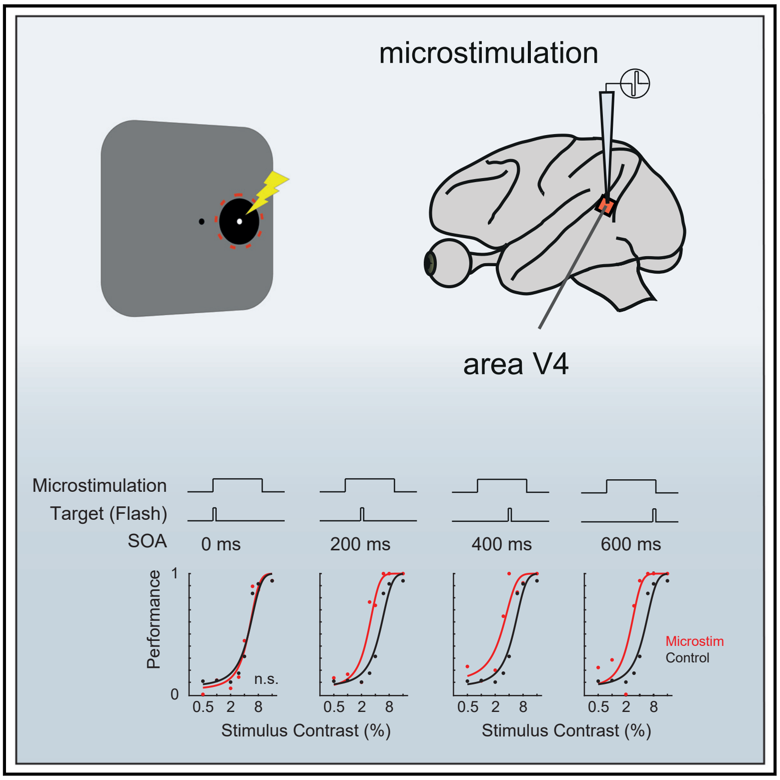

Authors

Ricardo Kienitz, Kleopatra Kouroupaki, Michael C. Schmid

Correspondence

kienitz@med.uni-frankfurt.de (R.K.), michael.schmid@unifr.ch (M.C.S.)

In brief

Kienitz et al. report that microstimulation of visual area V4 improves visual stimulus detection in macaque monkeys. This faciliatory effect extends to the other hemifield and is time dependent, consistent with dynamics of endogenous attention. These results highlight the critical role of V4 for visual processing and attention.

Highlights

- Microstimulation of visual area V4 improves visual stimulus detection
- Effects of V4 microstimulation extend to the other hemifield
- Microstimulation effects are time dependent and consistent with attention dynamics

Kienitz et al., 2022, Cell Reports *40*, 111392
September 20, 2022 © 2022 The Authors.
https://doi.org/10.1016/j.celrep.2022.111392

CellPress

Cell Reports

CellPress
OPEN ACCESS

Report

Microstimulation of visual area V4 improves visual stimulus detection

Ricardo Kienitz,[1,2,3,5,*] Kleopatra Kouroupaki,[2] and Michael C. Schmid[2,3,4,*]
[1]Epilepsy Center Frankfurt Rhine-Main, Center of Neurology and Neurosurgery, Goethe University, Schleusenweg 2-16, 60528 Frankfurt am Main, Germany
[2]Ernst Strüngmann Institute (ESI) for Neuroscience in Cooperation with Max Planck Society, Deutschordenstrasse 46, 60528 Frankfurt, Germany
[3]Institute of Neuroscience, Newcastle University, Framlington Place, Newcastle upon Tyne NE2 4HH, UK
[4]Department of Neuroscience and Movement Science, Faculty of Science and Medicine, University of Fribourg, Chemin du Musée 5, 1700 Fribourg, Switzerland
[5]Lead contact
*Correspondence: kienitz@med.uni-frankfurt.de (R.K.), michael.schmid@unifr.ch (M.C.S.)
https://doi.org/10.1016/j.celrep.2022.111392

SUMMARY

Neuronal activity in visual area V4 is well known to be modulated by selective attention, and there are reports on V4 lesions leading to attentional deficits. However, it remains unclear whether V4 microstimulation can elicit attentional benefits. To test this hypothesis, we performed local microstimulation in area V4 and explored its spatial and time dynamics in two macaque monkeys performing a visual detection task. Microstimulation was delivered via chronically implanted multi-electrode arrays. We found that microstimulation increases average performance by 35% and reduces luminance detection thresholds by −30%. This benefit critically depends on the onset of microstimulation relative to the stimulus, consistent with known dynamics of endogenous attention. These results show that local microstimulation of V4 can improve behavior and highlight the critical role of V4 for attention.

INTRODUCTION

Visual attention is well known to selectively modulate the processing of visual stimuli. In particular, it has been shown to modulate neuronal activity in midlevel visual area V4 (Maunsell, 2015; Moran and Desimone, 1985; Roe et al., 2012). Whether this attentional modulation arises directly from the activity of local V4 neurons or represents the influence of remote brain areas is not well understood. There is strong evidence that V4 attentional modulation might result from top-down feedback signals, in particular from the frontal eye field (FEF) in prefrontal cortex (see, e.g., Maunsell 2015 for a discussion). It has for example been shown that during selective attention oscillatory coupling in the gamma range is increased between FEF and V4 (Gregoriou et al., 2009). Furthermore, microstimulation of FEF can improve behavioral performance spatially selective (Moore and Fallah, 2001, 2004) and increase V4 responses in the receptive field (RF) reminiscent of the modulation produced by spatial attention (Armstrong et al., 2006; Moore and Armstrong, 2003). Lesioning FEF in turn can reduce the attention-related modulation of neuronal responses in V4 (Gregoriou et al., 2014).

While these findings suggest a prominent “control” function of prefrontal areas over V4 activity, they appear in contradiction to observations that lesioning V4 resulted in profound attentional deficits on the behavioral level (Gallant et al., 2000; Schiller, 1993, 1995; De Weerd et al., 2003), suggesting a critical role of V4 for attentional control and potentially a rather local source of its modulation (though the lesion may also influence remote areas). In contrast to these strong effects in lesion studies, surprisingly little is known about (attentional) benefits from V4 microstimulation. One study reported that V4 microstimulation can bias perceptual decisions during a disparity discrimination task (Shiozaki et al., 2012), while two studies found no effect on behavior in the detection of phosphenes or visual stimulus changes (Dagnino et al., 2015) and in a texture segregation task (Kerkoerle et al., 2014). Microstimulation of primary visual cortex even had a detrimental effect on behavior by delaying the execution of saccades and decreasing performance in visual detection tasks, which was interpreted as a masking or interference effect (Tehovnik and Slocum, 2005; Tehovnik et al., 2004, 2005).

We reasoned that the timing of microstimulation during task performance might play a critical role, in particular in experimental conditions that require the deployment of attentional resources. It is known that endogenous attention effects develop gradually over at least 100 ms after cue onset (Cheal and Lyon, 1991; Shepherd and Müller, 1989), which exceeds commonly used stimulation-target onset asynchronies (<100 ms). On the other hand, visual targets presented shortly after the end of microstimulation might be less likely to be detected, as microstimulation is known to induce a long-lasting neuronal inhibition (Logothetis et al., 2010). Thus, the unexplored time dynamics of

This is an open access article under the CC BY license (http://creativecommons.org/licenses/by/4.0/).

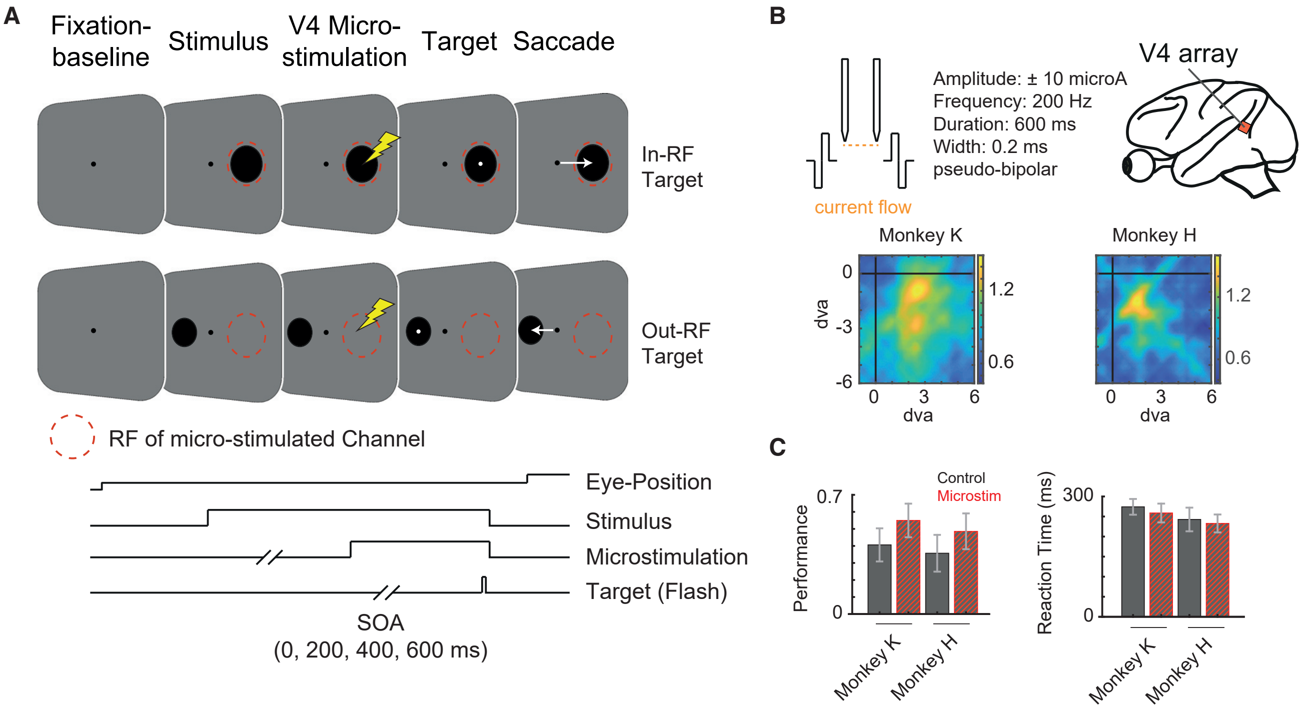

Figure 1. Experimental design

(A) Sketch depicting the paradigm. After a fixation baseline of 600 ms, a stimulus appeared either in the V4 receptive field or in a corresponding location on the other hemifield. This was followed by the onset of microstimulation. After either 0, 200, 400, or 600 ms, a target of varying contrast appeared.

(B) Microstimulation was performed in left hemispheres V4 with biphasic stimuli and a pseudo-bipolar stimulation regime that uses reversed pulse polarity between electrodes (depicted by sketch). Receptive fields of stimulated electrodes are displayed for monkey K (left) and H (right) in the lower panel.

(C) Left panel depicts average performance during detection trials across all target contrasts and locations without and with microstimulation (gray versus red) for monkey K and H (mean ± SEM). Right panel shows average reaction times for the same conditions. For a single target contrast and position 65.1 ± 1.0 and 68.1 ± 0.6 trials were included into the analysis in monkey K and H, respectively (mean ± SEM).

endogenous attention and microstimulation effects might have prevented the detection of a beneficial behavioral effect induced by microstimulation of V4 so far. We therefore systematically assessed the timing influence of V4 microstimulation in the range of known attentional dynamics while two macaque monkeys performed a visual detection task.

RESULTS

To investigate the effect of electrical microstimulation in V4 on visual perception, monkeys performed a visual detection task during which they had to report the presence of a small target of varying luminance contrast within a larger stimulus by executing a saccadic eye movement to the target. To control for spatial specificity, the stimulus and target could either appear within the RF of the V4 stimulation site (in-RF) or on a retinotopically corresponding location in the other hemifield (out-RF) (Figure 1A). Microstimulation was delivered between two neighboring electrodes of chronically implanted multi-electrode arrays (±10 μA, 200 Hz) and could precede the onset of a visual target by 0, 200, 400, or 600 ms (Figures 1A and 1B). To control behavior, detection trials were accompanied by catch trials during which no target appeared, and monkeys had to keep fixation throughout the trial. Performance in catch trials was generally high (93.5% in monkey K and 94.3% in monkey H across conditions), while corresponding false alarm rates were low and showed no systematic change with microstimulation (6.6% in monkey K and 5.7% in monkey H across conditions). Importantly, microstimulation also did not significantly change the rate of overt saccades or fixation breaks (see Table S1 for further details).

We found that microstimulation increased average performance across all target contrasts by 35.3% (from 40.6% ± 9.7% to 54.9% ± 9.9%) in monkey K and by 36.1% (from 35.7% ± 10.8% to 48.6% ± 10.5%) in monkey H. Corresponding average reaction times across all target contrasts decreased by −5.6% (from 274.1 ± 19.6 ms to 258.8 ± 23.4 ms) in monkey K and by −4.1% (from 242.6 ± 29.5 to 232.7 ± 22.3 ms) in monkey H (mean ± SEM; Figure 1C). However, as expected, behavioral performance strongly depended on target contrast (e.g., Figure 2). To further study the effect of microsimulation on behavior, we therefore constructed psychometric curves by fitting a logistic regression to the performance data (see STAR Methods for further details). Psychometric curves allow quantification of the detection thresholds, i.e., the target contrasts at which performance is 50%, and the rate of change around this threshold, i.e., the slope of the curve. Instead of comparing performance and reaction time values directly, we then quantified changes of these parameters of the psychometric curve.

CellPress
OPEN ACCESS

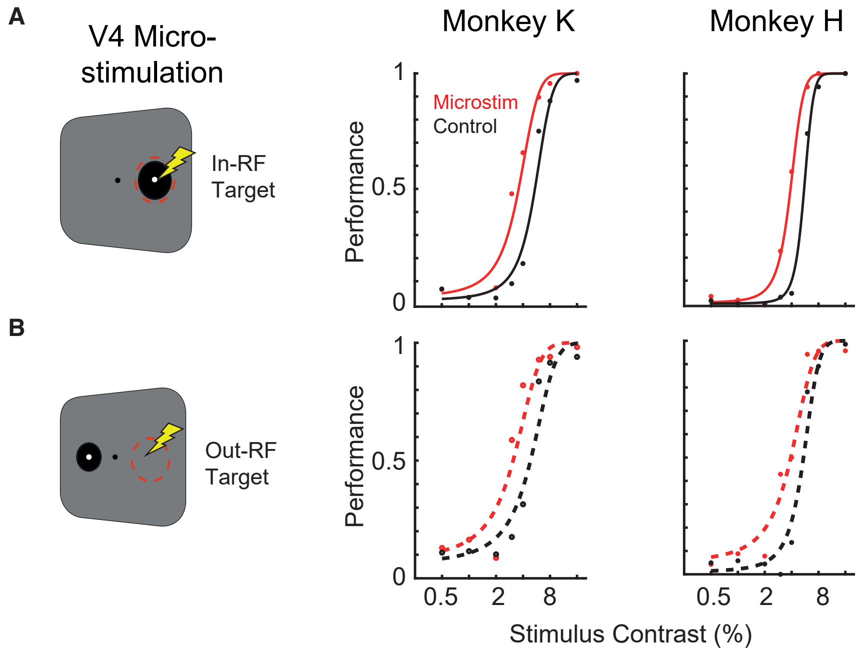

Figure 2. V4 microstimulation improves target detection

(A) Performance and psychometric curve fits for targets within the V4 receptive field as a function of target contrast for microstimulation (red) and control condition (black) for monkey K (left panel) and H (right panel), calculated across included trials (65.1 ± 1.0 and 68.1 ± 0.6 for a given target contrast and position in monkey K and H, respectively [mean ± SEM]).

(B) Same as (A) but for targets out of the receptive field (out-RF condition).

We found that microstimulation systematically and significantly lowered the detection threshold (Figure 2A) for targets within the RF (in-RF), consistent with a change in contrast gain known from attentional modulation of V4 firing (Reynolds et al., 2000). Specifically, the threshold decreased by −34.2% from 5.44 ± 0.19 to 3.58 ± 0.14 ($p = 1.1 \times 10^{-16}$, transformed likelihood ratio [TLR] test) in monkey K and by −29.4% from 5.53 ± 0.12 to 3.90 ± 0.12 ($p = 1.1 \times 10^{-16}$, TLR test) in monkey H. Microstimulation also had the tendency to increase the slope, with less consistent results across monkey though (+28.8% in monkey K, p = 0.032, TLR test, and +2.4% in monkey H, p = 0.864, TLR test).

Thus, V4 microstimulation enabled the monkey to detect lower contrast targets in the RF better compared with no-microstimulation controls, reminiscent of an attentional improvement.

Effects of V4 microstimulation extend to the other hemifield

Earlier studies focused on visual detection performance within the RF of the electrode at which microstimulation was applied (Dagnino et al., 2015; Shiozaki et al., 2012; Tehovnik and Slocum, 2005; Tehovnik et al., 2004), as attention as well as microstimulation can exert spatially specific effects (e.g., Salzman et al., 1992; Celebrini and Newsome 1995; Ditterich et al. 2003). On the other hand, visual attention can also act less spatially specific in the context of large attentional fields or feature-based attention, which both have been shown to also modulate neuronal activity in V4 (Hayden and Gallant, 2009; Maunsell and Treue, 2006; McAdams and Maunsell, 2000; Reynolds and Heeger, 2009; Zhou and Desimone, 2011). Further, it is known that V4 RFs can extend to the other hemifield, often covering corresponding retinotopic sites (Pigarev et al., 2001).

To test the spatial extent of V4 microstimulation, we aimed to test whether the beneficial effect of V4 microstimulation is also present when the visual target appeared far away from the stimulated cortical location. To this end, the visual stimulus was positioned on the isoeccentric ipsilateral position to the microstimulation site (out-RF condition), resulting in microstimulation and visual stimulation targeting opposite visual hemifields (Figure 1A). Quite surprisingly, we found that V4 microstimulation also significantly improved behavior of target detection in this out-RF condition (Figure 2B). Specifically, microstimulation decreased detection contrasts by −36.0% from 4.85 ± 0.20 to 3.10 ± 0.14 ($p = 8.5 \times 10^{-11}$, TLR test) in monkey K and by −27.9% from 5.43 ± 0.17 to 3.91 ± 0.18 ($p = 3.2 \times 10^{-10}$, TLR test). Again, as in the in-RF condition, changes of the psychometric function (PF) slope were less consistent (+41.3 % in monkey K, p = 0.009, and −12.9% in monkey H, p = 0.246, TLR test). Furthermore, there were no significant differences when directly comparing thresholds in-RF versus out-RF conditions (p = 0.67 and p = 0.955 in monkey K and p = 0.67 and p = 0.955 in monkey H, without and with microstimulation, respectively, TLR test).

Thus, the beneficial effect of V4 microstimulation appears to extend to spatially distant locations.

Facilitatory effect of microstimulation is time dependent

How attention aids stimulus detection depends very much on the timing between attentional cues and target onset. Previous work has established that endogenous attention develops gradually over at least 100 ms, while exogenous attention emerges rapidly within tens of milliseconds of cue onset (Cheal and Lyon, 1991; Shepherd and Müller, 1989).

To investigate this timing aspect of attention, we systematically varied the stimulus onset asynchrony (SOA) between onset of the microstimulation and the target. Specifically, the onset of the microstimulation could precede the onset of the target by either 0, 200, 400, or 600 ms with other parameters of the microstimulation being fixed.

When microstimulation and visual target onsets occurred in parallel (SOA = 0 ms), which due to conduction delays to V4, led the microstimulation to actually precede the target in the range of tens of milliseconds, no significant changes of thresholds were observable (p = 0.065 in monkey K and p = 0.113 in monkey H, TLR test, Figure 3A). However, when microstimulation preceded the onset of the target for longer periods,

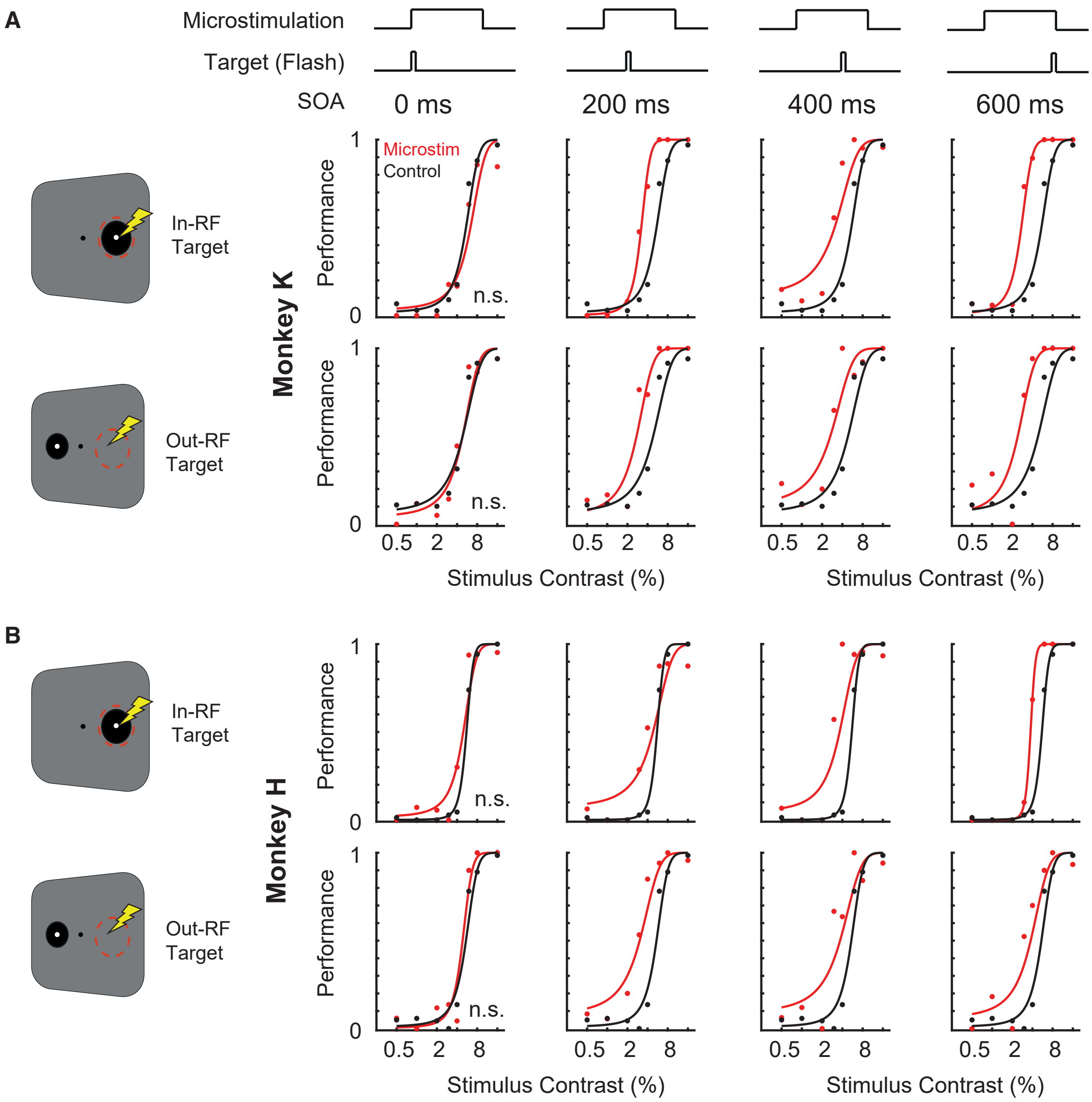

Figure 3. Behavioral performance as a function of microstimulation-target SOA

(A) Performance and psychometric curve fits for targets in the V4 RF (upper panels) and out of the RF (lower panels) as a function of target contrasts for different microstimulation-target SOAs (depicted in the upper row) for microstimulation (red) and control condition (black) for monkey K; n.s. denotes non-significance. Note the left shift of psychometric curves with microstimulation for higher SOA. Significance was assessed using the transformed likelihood ratio and chi-square distribution. An alternative approach based on 1,000 Monte Carlo simulations yielded similar results.

(B) Same as (A) but for monkey H.

thresholds decreased significantly. Specifically, the thresholds decreased by −40.8 ($p = 1.1 \times 10^{-16}$), −43.7 ($p = 1.9 \times 10^{-9}$), and −49.9 ($p = 1.1 \times 10^{-16}$) in monkey K for increasing SOAs (200, 400, and 600 ms, respectively; TLR test). In monkey H thresholds decreased by −12.8 ($p = 0.111$), −34.2 (p: 1.5×10^{-7}), and −32.3 ($p = 1.1 \times 10^{-16}$) for increasing SOAs (TLR test).

Interestingly, this time-dependent effect was again also observable for targets presented on the contralateral hemisphere (out-RF). Again, there was no significant change with microstimulation shortly preceding the target (SOA = 0 ms) in monkey K ($p = 0.976$) and H ($p = 0.061$). For longer SOAs, however, thresholds decreased by −43.6 ($p = 7.4 \times 10^{-11}$), −45.4 ($p = 8.6 \times 10^{-9}$), and −50.3 ($p = 8.8 \times 10^{-14}$) in monkey K and by −43.9 ($p = 2.2 \times 10^{-11}$), −31.9 ($p = 4.2 \times 10^{-5}$), and −30.9 ($p = 3.6 \times 10^{-5}$) in monkey H (SOA 200, 400, and 600 ms, respectively; TLR test). Changes to the slopes of the psychometric function were less consistent (see Table S2 for further details).

Thus, while a short SOA did not significantly alter detection thresholds, increasing the time of microstimulation preceding target presentation appeared to increase behavioral performance (Figures 3 and 4), consistent with the time course of endogenous attention. This effect was again not spatially

CellPress
OPEN ACCESS

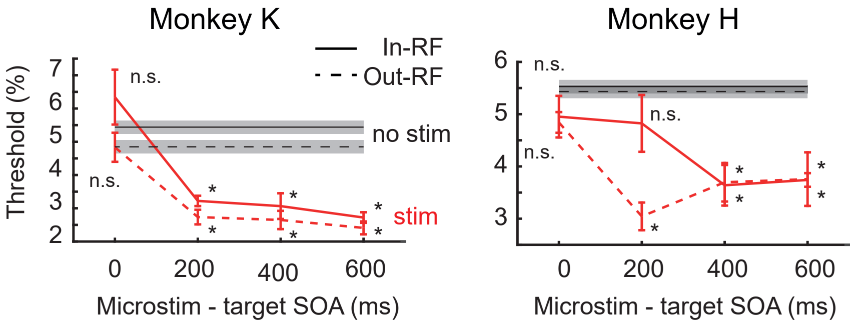

Figure 4. Time dynamics of the microstimulation effect

Left panel: mean thresholds for targets in (solid line) versus out of the receptive field (dashed line) for microstimulation (red) and control condition (black) as a function of microstimulation-target SOA (threshold ± SD). Asterisks denotes significance, n.s. non-significance. Right panel: same as left panel but for monkey H. SD was computed using a non-parametric bootstrap method with 100 repetitions.

confined to the location of cortical microstimulation but extended to the contralateral hemisphere (Figures 3 and 4).

DISCUSSION

Our results show that electrical microstimulation of V4 can improve visual detection. The observed performance gain extended to spatial locations distant to the stimulated RF. Furthermore, the beneficial effect depended on the time of the microstimulation preceding the presentation of the visual target. In the following, we compare these findings to earlier V4 microstimulation studies and provide an interpretation of the observed effects in terms of attentional mechanisms.

Lack of consistent behavioral effects across V4 stimulation studies

Despite the fact that electrical microstimulation is one of the most established methods in neuroscience and neurology, there are to date only less than a handful of studies focusing on its application in V4. Moreover, the results from existing microstimulation studies differ widely in range. Shiozaki et al. used microstimulation (40 μA, 200 Hz, biphasic pulses) during a fine disparity discrimination task (Shiozaki et al., 2012). By stimulating *during* the presentation of visual stimuli (1.5 s), they could bias the monkey's choice toward the preferred "choice" (far versus near) of the stimulated multi-unit. This was evident in a left or right shift of the psychometric function without changes to its slope. Two other studies however reported null results (Dagnino et al., 2015; Kerkoerle et al., 2014). Dagnino et al. first examined the effect of very short V4 microstimulation (three to four pulses, amplitude at 50% of phosphene detection threshold, ca. 25 μA, 200 Hz, biphasic pulses) on the detection of phosphenes induced by V1 microstimulation and found no specific effect of V4 microstimulation (Dagnino et al., 2015). In a second experiment, they tested the effect of V4 microstimulation in a distributed attention task. Using a train of 100 ms microstimulation immediately preceding the onset of the target (dimming of a bar), they again found no significant effect of V4 microstimulation. This might be explained by the fact that microstimulation can lead to an artificial long-lasting inhibition (Logothetis et al., 2010), which can reduce detection performance of stimuli presented shortly after the end of microstimulation. Importantly, in our case and in the study by Shiozaki et al., microstimulation overlapped with target presentation, which likely contributed to the positive effects. Not finding a microstimulation effect in the study by Kerkoerle et al. can in turn be explained by the behavioral accuracy already being close to saturation in non-stimulation conditions, likely preventing further improvement by microstimulation (Kerkoerle et al., 2014). The use of distinct stimulation regimes ("monopolar" by Shiozaki et al. versus bipolar by Kerkoerle et al. and Roelfsema et al.) might further have contributed to differences in effect sizes. In our study, we combined low amplitudes with a pseudo-bipolar regime where reversed pulse polarity at neighboring electrodes was applied to limit current spread (see also STAR Methods).

Interpretation in the context of attention mechanisms

Microstimulation in the distant frontal eye field can mimic attentional effects in V4 and behavior (Armstrong et al., 2006; Moore and Armstrong, 2003; Moore and Fallah, 2001, 2004), which led to the hypothesis that attentional signals might arise in higher areas, such as FEF, and be propagated to V4 by feedback signals (see, e.g., Maunsell 2015 for a discussion also including superior colliculus and posterior parietal cortex).

In contrast to this feedback hypothesis, V4 lesion studies appear to point to a rather local source by reporting profound attentional deficits (Gallant et al., 2000; Schiller, 1993, 1995; De Weerd et al., 2003). More specifically, lesioning V4 caused an *increase* of the threshold (i.e., a right shift of psychometric curve) of the luminance- and color-contrast detection (Schiller, 1993), which—according to the normalization model of attention—is consistent with affecting a large attentional field (i.e., a loss in contrast gain) (Reynolds and Heeger, 2009).

Our results appear consistent with the V4 lesion effects as microstimulating this area resulted in a 30% behavioral benefit. This was evident in a corresponding *decrease* of contrast detection threshold. Interestingly, the facilitatory effect of microstimulation on target detection was only present when microstimulation preceded visual target onset by at least 200 ms. This time-sensitive nature of the microstimulation effect is further consistent with the dynamics of endogenous attention that gradually increase over hundreds of milliseconds (Cheal and Lyon, 1991; Shepherd and Müller, 1989). In turn, the missing effect at 0 SOA, where microstimulation effectively preceded the stimulus evoked signal by tens of milliseconds due to conduction delays, speaks against exogenous-like attentional effects that are known to act within tens of milliseconds (Cheal and Lyon, 1991; Shepherd and Müller, 1989). It further argues against a simple increase in neuronal excitability as this would be expected to be effective without significant delays.

Could the effect alternatively also be explained by the microstimulation acting as a nonspecific alerting cue? Such alerting or neutral cues act quickly and improve reaction times that are spatially unspecific by carrying temporal information about when the target will appear. However, in our case a high amount of catch trials and a mixture of different SOAs essentially prevented a predictive value of the stimulation regarding target timing. Alerting cures further typically show an increase in effect size with increasing SOAs as expectancy builds up (Niemi and Näätänen, 1981; Posner and Cohen, 1984; Weinbach and Henik, 2012), which is in contrast to the stable RT effect at longer SOAs in our data. Lastly, peripheral cues that act as alerting cues (but do not necessarily carry spatial information about a target) show an inhibition of return effect, an inhibitory effect after ca. 200 ms during exogenous attention (Maylor and Hockey, 1985; Posner and Cohen, 1984), which is not present in our data. Taken together, these points speak against the microstimulation acting as a nonspecific alerting cue; rather an interpretation in the context of endogenous visual attention seems more plausible at this point.

The extension of our microstimulation effects to the contralateral hemifield appear somewhat unexpected in the context of the classical retinotopic organization of V4 and studies reporting dominantly local and sparse suprathreshold neuronal activation, i.e., spiking, close to the stimulating electrodes tip (Histed et al., 2009; Stoney et al., 1968). By using low amplitudes and a pseudo-bipolar stimulation regime, we further spatially limited neuronal activation. The length of microstimulation was also shown to have only little effect on the local activation pattern (Histed et al., 2009). Other studies found that microstimulation can—by using high stimulation amplitudes (up to 10–100 times higher than in our setup)—in principle also lead to widespread subthreshold activity (Logothetis et al., 2010; Sultan et al., 2007; Tolias et al., 2005).

There are however reports showing that V4 RFs can in fact cross the vertical meridian and typically cover isoeccentric locations in both hemifields (Pigarev et al., 2001). Thus, a spatially limited neuronal activation in V4 could in principle lead to bilateral effects. Alternatively, the results would also be in line with the induction of large attentional fields or feature-based attention, which both have been shown to act less spatially specific and modulate neuronal activity in V4 (Hayden and Gallant, 2009; Maunsell and Treue, 2006; McAdams and Maunsell, 2000; Reynolds and Heeger, 2009; Zhou and Desimone, 2011). The notion of large attentional fields would further be consistent with V4 microstimulation and lesion both modulating the contrast instead of response gain (Reynolds and Heeger, 2009; Schiller, 1993).

In summary, our results show that local V4 microstimulation can improve behavior in a simple visual detection task mimicking visual attention. In the face of widespread attentional signals in the brain, it highlights the relevance of V4 for modulating the processing of visual information.

Limitations of the study

First, due to ethical reasons, the number of subjects (macaque monkeys) was limited to two in our study. Hence no statistical testing of results across subjects was possible. Secondly, we controlled for spatial selectivity of the microstimulation effect by introducing a control stimulus on the isoeccentric location of the V4 RF where microstimulation was delivered. However, assessment of spatial selectivity would have benefited from additional control target locations, e.g., in the upper visual field. Third, stimulating for a prolonged period of time did not allow for analysis of the neural signal that was recorded in parallel. Further studies might leverage a combination of different recording techniques (e.g., optical imaging and microstimulation or electrophysiology and optogenetics) to assess neural activity while stimulating. Lastly, we stimulated locally in V4 given its prominent electrophysiological attentional modulation. However, as discussed above, other areas likely significantly contribute to the behavioral attentional effect. Future studies may combine recording and stimulation techniques in a set of potentially involved areas to disentangle their interaction and contribution to behavior during visual attention.

STAR★METHODS

Detailed methods are provided in the online version of this paper and include the following:

- KEY RESOURCES TABLE
- RESOURCE AVAILABILITY
  - Lead contact
  - Materials availability
  - Data and code availability
- EXPERIMENTAL MODEL AND SUBJECT DETAILS
- METHOD DETAILS
  - Behavioral paradigm
  - Neurophysiological setup and microstimulation
- QUANTIFICATION AND STATISTICAL ANALYSIS
  - Data analysis
  - Psychometric functions and statistics

SUPPLEMENTAL INFORMATION

Supplemental information can be found online at https://doi.org/10.1016/j.celrep.2022.111392.

ACKNOWLEDGMENTS

This work was supported by DFG Emmy Noether grant SCHM 2806/1-1, ERC OptoVision 637638, and SNF grant BSET-0_201532 to M.C.S.

AUTHOR CONTRIBUTIONS

Conceptualization, R.K. and M.C.S.; methodology, R.K., K.K., and M.C.S.; software, R.K. and K.K.; formal analysis, R.K.; investigation, R.K. and K.K.; writing – original draft, R.K. and M.C.S.; writing – review & editing, R.K., K.K., and M.C.S.; supervision, M.C.S.

DECLARATION OF INTERESTS

The authors declare no competing interests.

Received: March 14, 2022
Revised: June 30, 2022
Accepted: August 29, 2022
Published: September 20, 2022

REFERENCES

Armstrong, K.M., Fitzgerald, J.K., and Moore, T. (2006). Changes in visual receptive fields with microstimulation of frontal cortex. Neuron *50*, 791–798.

Celebrini, S., and Newsome, W.T. (1995). Microstimulation of extrastriate area MST influences performance on a direction discrimination task. J. Neurophysiol. *73*, 437–448.

Cheal, M., and Lyon, D.R. (1991). Central and peripheral precuing of forced-choice discrimination. Q. J. Exp. Psychol. *43*, 859–880.

Dagnino, B., Gariel-Mathis, M.A., and Roelfsema, P.R. (2015). Microstimulation of area V4 has little effect on spatial attention and on perception of phosphenes evoked in area V1. J. Neurophysiol. *113*, 730–739.

Ditterich, J., Mazurek, M.E., and Shadlen, M.N. (2003). Microstimulation of visual cortex affects the speed of perceptual decisions. Nat. Neurosci. *6*, 891–898.

Fiorani, M., Azzi, J.C.B., Soares, J.G.M., and Gattass, R. (2014). Automatic mapping of visual cortex receptive fields: a fast and precise algorithm. J. Neurosci. Methods *221*, 112–126.

Gallant, J.L., Shoup, R.E., and Mazer, J.A. (2000). A human extrastriate area functionally homologous to macaque V4. Neuron *27*, 227–235.

Gregoriou, G.G., Gotts, S.J., Zhou, H., and Desimone, R. (2009). High-Frequency, long-range coupling between prefrontal and visual cortex during attention. Science *324*, 1207–1210.

Gregoriou, G.G., Rossi, A.F., Ungerleider, L.G., and Desimone, R. (2014). Lesions of prefrontal cortex reduce attentional modulation of neuronal responses and synchrony in V4. Nat. Neurosci. *17*, 1003–1011.

Hao, Y., Riehle, A., and Brochier, T.G. (2016). Mapping horizontal spread of activity in monkey motor cortex using single pulse microstimulation. Front. Neural Circuits *10*, 104.

Hayden, B.Y., and Gallant, J.L. (2009). Combined effects of spatial and feature-based attention on responses of V4 neurons. Vision Res. *49*, 1182–1187.

Histed, M.H., Bonin, V., and Reid, R.C. (2009). Direct activation of sparse, distributed populations of cortical neurons by electrical microstimulation. Neuron *63*, 508–522.

van Kerkoerle, T., Self, M.W., Dagnino, B., Gariel-Mathis, M.-A., Poort, J., van der Togt, C., and Roelfsema, P.R. (2014). Alpha and gamma oscillations characterize feedback and feedforward processing in monkey visual cortex. Proc. Natl. Acad. Sci. USA *111*, 14332–14341.

Logothetis, N.K., Augath, M., Murayama, Y., Rauch, A., Sultan, F., Goense, J., Oeltermann, A., and Merkle, H. (2010). The effects of electrical microstimulation on cortical signal propagation. Nat. Neurosci. *13*, 1283–1291.

Maunsell, J.H.R. (2015). Neuronal mechanisms of visual attention. Annu. Rev. Vis. Sci. *1*, 373–391.

Maunsell, J.H., and Treue, S. (2006). Feature-based attention in visual cortex. Trends Neurosci. *29*, 317–322.

Maylor, E.A., and Hockey, R. (1985). Inhibitory component of externally controlled covert orienting in visual space. J. Exp. Psychol. Hum. Percept. Perform. *11*, 777–787.

McAdams, C.J., and Maunsell, J.H. (2000). Attention to both space and feature modulates neuronal responses in macaque area V4. J. Neurophysiol. *83*, 1751–1755.

Moore, T., and Armstrong, K.M. (2003). Selective gating of visual signals by microstimulation of frontal cortex. Nature *421*, 370–373.

Moore, T., and Fallah, M. (2001). Control of eye movements and spatial attention. Proc. Natl. Acad. Sci. USA *98*, 1273–1276.

Moore, T., and Fallah, M. (2004). Microstimulation of the frontal eye field and its effects on covert spatial attention. J. Neurophysiol. *91*, 152–162.

Moran, J., and Desimone, R. (1985). Selective attention gates visual processing in the extrastriate cortex. Science *229*, 782–784.

Niemi, P., and Näätänen, R. (1981). Foreperiod and simple reaction time. Psychol. Bull. *89*, 133–162.

Pigarev, I.N., Nothdurft, H.C., and Kastner, S. (2001). Neurons with large bilateral receptive fields in monkey prelunate gyrus. Exp. Brain Res. *136*, 108–113.

Posner, M.I., and Cohen, Y. (1984). Components of visual orienting. In Attention and Performance, pp. 531–556.

Prins, N., and Kingdom, F.A.A. (2018). Applying the model-comparison approach to test specific research hypotheses in psychophysical research using the Palamedes toolbox. Front. Psychol. *9*, 1250–1314.

Qiao, S., Brown, K.A., Orsborn, A.L., Ferrentino, B., and Pesaran, B. (2016). Development of semi-chronic microdrive system for large-scale circuit mapping in macaque mesolimbic and basal ganglia systems. In 2016 38th Annual International Conference of the IEEE Engineering in Medicine and Biology Society (EMBC) (IEEE), pp. 5825–5828.

Qiao, S., Sedillo, J.I., Brown, K.A., Ferrentino, B., and Pesaran, B. (2020). A causal network analysis of neuromodulation in the mood processing network. Neuron *107*, 972–985.e6.

Reynolds, J.H., and Heeger, D.J. (2009). The normalization model of attention. Neuron *61*, 168–185.

Reynolds, J.H., Pasternak, T., and Desimone, R. (2000). Attention increases sensitivity of V4 neurons. Neuron *26*, 703–714.

Roe, A.W., Chelazzi, L., Connor, C.E., Conway, B.R., Fujita, I., Gallant, J.L., Lu, H., and Vanduffel, W. (2012). Toward a unified theory of visual area V4. Neuron *74*, 12–29.

Salzman, C.D., Murasugi, C.M., Britten, K.H., and Newsome, W.T. (1992). Microstimulation in visual area MT: effects on direction discrimination performance. J. Neurosci. *12*, 2331–2355.

Schiller, P.H. (1993). The effects of V4 and middle temporal (MT) area lesions on visual performance in the rhesus monkey. Vis. Neurosci. *10*, 717–746.

Schiller, P.H. (1995). Effect of lesions in visual cortical area V4 on the recognition of transformed objects. Nature *376*, 342–344.

Shapcott, K.A., Schmiedt, J.T., Kouroupaki, K., Kienitz, R., Lazar, A., Singer, W., and Schmid, M.C. (2020). Reward-related suppression of neural activity in macaque visual area v4. Cereb. Cortex. *30*, 4871–4881.

Shepherd, M., and Müller, H.J. (1989). Movement versus focusing of visual attention. Percept. Psychophys. *46*, 146–154.

Shiozaki, H.M., Tanabe, S., Doi, T., and Fujita, I. (2012). Neural activity in cortical area V4 underlies fine disparity discrimination. J. Neurosci. *32*, 3830–3841.

Stoney, S.D., Thompson, W.D., and Asanuma, H. (1968). Excitation of pyramidal tract cells by intracortical microstimulation: effective extent of stimulating current. J. Neurophysiol. *31*, 659–669.

Sultan, F., Augath, M., and Logothetis, N. (2007). BOLD sensitivity to cortical activation induced by microstimulation: comparison to visual stimulation. Magn. Reson. Imaging *25*, 754–759.

Tehovnik, E.J., and Slocum, W.M. (2005). Microstimulation of V1 affects the detection of visual targets: manipulation of target contrast. Exp. Brain Res. *165*, 305–314.

Tehovnik, E.J., Slocum, W.M., and Schiller, P.H. (2004). Microstimulation of V1 delays the execution of visually guided saccades. Eur. J. Neurosci. *20*, 264–272.

Tehovnik, E.J., Slocum, W.M., and Schiller, P.H. (2005). Delaying visually guided saccades by microstimulation of macaque V1: spatial properties of delay fields. Eur. J. Neurosci. *22*, 2635–2643.

Tolias, A.S., Sultan, F., Augath, M., Oeltermann, A., Tehovnik, E.J., Schiller, P.H., and Logothetis, N.K. (2005). Mapping cortical activity elicited with electrical microstimulation using fMRI in the macaque. Neuron *48*, 901–911.

De Weerd, P., Desimone, R., and Ungerleider, L.G. (2003). Generalized deficits in visual selective attention after V4 and TEO lesions in macaques. Eur. J. Neurosci. *18*, 1671–1691.

Weinbach, N., and Henik, A. (2012). Temporal orienting and alerting - the same or different? Front. Psychol. *3*, 236.

Zhou, H., and Desimone, R. (2011). Feature-based attention in the frontal eye field and area V4 during visual search. Neuron *70*, 1205–1217.

STAR★METHODS

KEY RESOURCES TABLE

REAGENT or RESOURCE	SOURCE	IDENTIFIER
Experimental models: Organisms/strains		
Macaca mulatta	Public Health England, Porton Down, UK	Monkey K, Monkey H
Software and algorithms		
MATLAB	The MathWorks	https://www.mathworks.com/products/matlab.html
PALAMEDES toolbox for MATLAB	Prins and Kingdom, 2018	https://www.palamedestoolbox.org
Other		
Infrared video eye tracking system	EyeLink	https://www.sr-research.com/products/
Data Acquisition and Stimulation Systems	Blackrock Microsystems	http://blackrockmicro.com/neuroscience-research-products/

RESOURCE AVAILABILITY

Lead contact

Further information and requests for resources should be directed to and will be fulfilled by the lead contact, Ricardo Kienitz (kienitz@med.uni-frankfurt.de).

Materials availability

This study did not generate new unique reagents.

Data and code availability

- Data reported in this paper will be shared by the lead contact upon request.
- This paper does not report original code.
- Any additional information required to reanalyze the data reported in this paper is available from the lead contact upon request.

EXPERIMENTAL MODEL AND SUBJECT DETAILS

We trained two adult healthy male rhesus monkeys (Macaca mulatta, monkey K and H) on a simple visual detection task (see below). All procedures were approved by the Regierungspräsidium Darmstadt and carried out in accordance with the applicable laws and regulations. The monkeys were group peer-housed in enriched environments and with access to outdoor space. All surgeries were carried out aseptically under gas anesthesia using standard techniques including per-surgical analgesia and monitoring. Animals received controlled access to fluids during experimental periods to ensure motivation for the cognitive experiments in accordance with regulations. Each monkey was implanted with a titanium-made head-immobilization implant, a Blackrock multi-electrode array (“Utah-array”) including a connector plug (Blackrock Microsystems, Hannover, Germany) and a recording chamber. Throughout the study animal welfare was monitored by veterinarians, technicians and scientists.

METHOD DETAILS

Behavioral paradigm

During all the experiments eye movements were tracked using an infrared eye tracking system at a sampling rate of 500 Hz (EyeLink 1000, SR research, Ottawa, ON, Canada). Stimuli were presented on a Samsung 2233RZLCD screens (120 Hz refresh rate, 1680x1050 resolution, viewing distance was 77 cm for monkey K and 86 cm for monkey H). Stimulus presentation and monkey behavior during the experiments were controlled and monitored using MonkeyLogic.

All stimuli were shown on a gray background (50%). The trial was initiated by display of a small central dot that the monkey had to fixate within 5000 ms (fixation radius of 0.8 dva). Whenever the eye position left the fixation window (except for after target presentation) the trial was aborted, and the next trial initiated after 500 ms.

After acquiring fixation, fixation had to be held for 600 ms. Then, a single black disk was displayed either in the V4 receptive field (right hemisphere, in-RF condition) or on the corresponding location of the left hemifield (out-RF condition) for a minimum of 300 ms. After a randomized period between 0 and 400 ms electrical microstimulation was applied to area V4 via the implanted Utah array in the left hemisphere for 600 ms (see below for details on the microstimulation). With a relative delay either 0, 200, 400 or 600 ms to the microstimulation onset of (chosen randomly between the concrete delays) a small target was displayed in the center of the disk (left or right disk chosen randomly). Note, that at 0 SOA microstimulation in fact preceded the onset of the target by tens of milliseconds due to conduction delays. Target contrasts were randomly chosen from the following distribution: 0.5%, 1%, 2%, 3%, 4%, 6%, 8%, 16%. After target presentation monkeys had to respond within 650 ms by executing a saccade toward the target location and keep fixation there for at least 50 ms. Maximal duration of an allowed saccade was set to 200 ms to encourage only direct eye movements to the target. To encourage a conservative response behavior and suppress early responses, a high rate of catch trials was chosen (ca. 50%). During these no target was presented, and the monkey had to keep fixation for a total of 1100 ms after stimulus onset (equaling the sum of maximum delays of randomized periods during detection trials). Monkeys received a juice reward for correct trials.

The stimulus positions were chosen such that one fell into the V4 receptive field in the right visual hemifield while the other was positioned on the corresponding location of the other hemifield. Positions were [4,-1] and [-4,-1] for monkey K and [1.6,-1.5] and [-1.6,-1.5] for monkey H ([x,y] in dva relative to fixation spot).

Data were recorded during multiple sessions per monkey and eventually pooled across sessions for analysis purposes (7 sessions in monkey K, 6 sessions in monkey H).

Neurophysiological setup and microstimulation

Monkeys were implanted with 64 channel Blackrock multi-microelectrode "Utah" arrays (Blackrock Microsystems, Hannover, Germany) in the left hemisphere's area V4 (prelunate gyrus) and V1 (primary visual cortex). Electrodes had lengths of either 0.6 or 1 mm arranged in alternating sets of two rows of short and long electrodes. Each electrode was 400 μm away from its neighboring electrodes. Reference wires were inserted over parietal cortex and cerebellum. Neural data was recorded at a sampling rate of 30 kHz using the Blackrock Microsystems Cerebus system. In this study, neural data was not analyzed. Electrical microstimulation was delivered to two neighboring electrodes within the V4 array using the Blackrock CereStim R96 device. Electrodes were chosen due to stimulus coverage of their receptive field location which were computed based on multi-unit activity (see below). Thus, the in-RF visual target was displayed within the receptive fields of the stimulated electrodes. We therefore stimulated the same population of neurons in V4 that were also excited by the target. The control target (out-RF) was displayed at the corresponding site of the other hemifield thus being outside of the receptive field. The Blackrock CereStim R96 device allows for monopolar stimulation where the current flows toward the common ground (ground plane of the stimulator). Monopolar microstimulation regimes are commonly used (e.g. Tehovnik et al. 2004; Tehovnik and Slocum 2005; Shiozaki et al., 2012; Armstrong et al. 2006; Moore and Armstrong 2003) and have been shown to elicit very local neural and behavioral effects using Utah arrays, even with a distant common ground (Hao et al., 2016). However, to further limit the current flow in our setup, we used – besides low stimulation amplitudes and biphasic, pseudo-bipolar stimulation regime, where two neighboring electrodes are stimulated using reversed pulse polarity, e.g. cathodic at one channel and anodic at the neighboring channel during the first phase and vice versa during the second phase of the stimulus (Figure 1) (Qiao et al., 2016, 2020). This confines the current flow between the two neighboring electrodes. However, potential imbalances between the two pulses will flow toward the common ground. Microstimulation pulses had an amplitude of ±10 μA, a width of 0.2 ms and were applied for 600 ms with a frequency of 200 Hz.

QUANTIFICATION AND STATISTICAL ANALYSIS

Data analysis

All data were processed and analyzed using custom-written code for MATLAB (MathWorks, Inc.) and the PALAMEDES toolbox for MATLAB (Prins and Kingdom, 2018). Performance was computed as the proportion of correct trials relative to total trials. The number of total trials was the sum of correct trials, wrong trials (monkey did not respond to a target or chose a wrong target location) and false alarms. False alarms were defined as a saccade response to a target location before it was actually displayed. A catch trial was considered wrong if the monkey saccaded to a target location. Fixation breaks ended the trial immediately. For a single target contrast and position 65.1 ± 1.0 and 68.1 ± 0.6 trials were included into the analysis in monkey K and H, respectively (correct trials, wrong trials and false alarms; average across conditions). For a given catch trial condition 306.5 ± 2.9 and 347.8 ± 4.3 trials were recorded in monkey K and H, respectively.

Receptive fields in Figure 1 were computed using a bar mapping method as used by (Fiorani et al., 2014) based on multi-unit activity (see (Shapcott et al., 2020) for further details).

Psychometric functions and statistics

To compute psychometric curves, we fit a logistic psychometric function to the data using a Maximum Likelihood criterion and the Nelder-Mead Simplex method to find the maximum in the likelihood function (PAL_PFML_Fit function of the Palamedes toolbox). This way, parameters were computed for the threshold and slope of the curve. Standard deviations of the threshold and slope values were computed using a non-parametric bootstrap method with 100 repetitions (PAL_PFML_BootstrapNonParametric function of Palamedes toolbox).

Statistical comparison between psychometric functions was performed by computing the transformed likelihood ratio (TLR) ([-2 x ln(Likelihood(model1 model)/Likelihood(model2)]) of two fit models (PAL_PFLR_ModelComparison function of the Palamedes toolbox). p values were then based on the theoretical chi-square distribution. An alternative approach using 1000 Monte Carlo simulations of one condition and assessing the proportion of stimulated TLR exceeding the TLR of the other condition achieved similar results. For comparing rates of fixation breaks after onset of microstimulation a Chi-square test was used. If not stated otherwise summary statistics are reported as the mean ± standard deviation (SD).

Cell Reports, Volume *40*

Supplemental information

Microstimulation of visual area V4 improves visual stimulus detection

Ricardo Kienitz, Kleopatra Kouroupaki, and Michael C. Schmid

Supplementary Information

Catch trial performance				
	No stim		Stim	
	in-RF	out-RF	in-RF	out-RF
Monkey K	97.1%	92.3%	98.1%	86.3%
Monkey H	97.9%	90.3%	97.5%	91.3%
Change of fixation breaks with microstimulation				
	in-RF	p	out-RF	p
Monkey K	21.4%	0.38	-8.6%	0.67
Monkey H	28.6%	0.67	5.6 %	0.89

Table S1. Additional control analyses, related to Figure 1.
Note that fixation breaks showed no consisted or significant change with microstimulation (Chi-square test).

Thresholds – in-RF									
		SOA 0 ms		SOA 200 ms		SOA 400 ms		SOA 600 ms	
	No stim	Stim	p	Stim	p	Stim	p	Stim	p
Monkey K	5.44 ± 0.19	6.34 ± 0.74	0.064	3.22 ± 0.16	$1.1x10^{-16}$	3.07 ± 0.34	$1.9x10^{-9}$	2.72 ± 0.17	$1.1x10^{-16}$
Monkey H	5.53 ± 0.12	4.95 ± 0.42	0.113	4.82 ± 0.62	0.111	3.64 ± 0.39	$1.5x10^{-7}$	3.74 ± 0.15	$1.1x10^{-16}$
Thresholds – out-RF									
		SOA 0 ms		SOA 200 ms		SOA 400 ms		SOA 600 ms	
	No stim	Stim	p	Stim	p	Stim	p	Stim	p
Monkey K	4.85 ± 0.20	4.8345 ± 0.40	0.975	2.74 ± 0.21	$7.4x10^{-11}$	2.65 ± 0.29	$8.6x10^{-9}$	2.41 ± 0.21	$8.8x10^{-14}$
Monkey H	5.43 ± 0.19	4.84 ± 0.18	0.061	3.04 ± 0.28	$2.2x10^{-11}$	3.69 ± 0.35	$4.2x10^{-5}$	3.76 ± 0.52	$3.6x10^{-5}$
Slopes – in-RF									
		SOA 0 ms		SOA 200 ms		SOA 400 ms		SOA 600 ms	
	No stim	Stim	p	Stim	p	Stim	p	Stim	p
Monkey K	75.59 ± 10.51	54.42 ± 18.57	0.059	188.12 ± 44.59	$5.8x10^{-5}$	67.17 ± 24.66	0.544	179.57 ± 50.18	0.0001
Monkey H	143.54 ± 15.44	83.38 ± 36.66	0.087	52.87 ± 25.02	$1.5x10^{-8}$	84.09 ± 40.32	0.004	311.26 ± 75.02	0.006
Slopes – out-RF									
		SOA 0 ms		SOA 200 ms		SOA 400 ms		SOA 600 ms	
	No stim	Stim	p	Stim	p	Stim	p	Stim	p
Monkey K	55.06 ± 8.51	65.49 ± 19.97	0.353	110.35 ± 24.48	0.002	80.21 ± 29.68	0.115	123.36 ± 26.89	0.002
Monkey H	85.14 ± 12.63	115.07 ± 25.54	0.109	80.36 ± 43.89	0.771	62.18 ± 18.21	0.079	73.94 ± 35.69	0.473

Table S2. Changes of threshold and slope as a function of microstimulation-target SOA, related to Figure 3.
